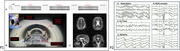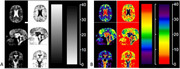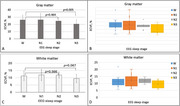# Impact of Sleep on Extracellular Space in Human Brains: A Confirmation Study with Simultaneous Sodium MRI and EEG

**DOI:** 10.1002/alz70856_101338

**Published:** 2025-12-25

**Authors:** Kennedy Watson, Xingye Chen, Ying‐Chia Lin, Simon Henin, Nahbila‐Malikha Kumbella, Justin Quimbo, Zena Rockowitz, Yulin Ge, Arjun V. Masurkar, Anli Liu, Yvonne W. Lui, Yongxian Qian

**Affiliations:** ^1^ New York University Grossman School of Medicine, New York, NY, USA; ^2^ NYU Grossman School of Medicine, New York, NY, USA; ^3^ Alzheimer's Disease Research Center, New York University Langone Health, New York, NY, USA

## Abstract

**Background:**

Sleep was associated with an increased extracellular volume fraction (ECVF) in mice and enhanced cerebrospinal fluid (CSF) clearance of neurotoxic amyloid‐beta (Aβ) proteins – a hallmark of Alzheimer's disease (AD). Such a beneficiary impact of sleep is however difficult to study on humans due to the lack of non‐invasive imaging techniques. Last year (2024), we were, with unique sodium MRI and MRI‐compatible EEG, able to study a cohort of healthy subjects and found a decrease, instead of increase, in ECVF during sleep. To confirm such an unexpected finding, here we report a study on a different cohort of healthy subjects using the same technologies as in the last‐year study, i.e., simultaneous measurements of ECVF by sodium (^23^Na) MRI and sleep by MRI‐compatible EEG.

**Method:**

This study (Figure 1) was performed on 30 cognitively normal human subjects (age 25–87 years, 22 females, 8 males), with approved IRB and signed consent. Each subject underwent a 90‐min sodium MRI (Siemens Prisma, 3T) with a dual‐tuned (^1^H‐^23^Na) birdcage head coil (QED, Cleveland, OH) and a continuous recording of MR‐compatible EEG (Brain Vision, 32 channels). MRI scans consist three segments, each of 16min long with a 2‐min gap in between for artifact‐free EEG recording. Sleep was scored to five stages (wake, N1, N2, N3, and REM) according to AASM standards (V2.6, 2020). The pulse sequence was custom‐developed twisted projection imaging (TPI). ECVF was quantified voxel‐by‐voxel using a two‐compartment model of intra‐ and extra‐cellular spaces.

**Result:**

Figure 1 (P2) shows representatives of our EEG waveforms from our study subjects at different sleep stages. Figure 2 presents typic maps of ECVF from an individual subject, while Figure 3 summarizes ECVF from all of the subjects studied. Overall, ECVF changed during sleep in both white and gray matter regions of the brain; decreasing statistically significant in gray matter regions during N3 (*p* = 0.005) but not in N2 (*p* = 0.464) and not in white matter regions during N3 (*p* = 0.067).

**Conclusion:**

We found ECVF to decrease in slow wave sleep (N3), confirming our previous results in a different cohort of subjects but contradicting previous animal studies. This prompts further investigation of physiological importance of sleep.